# Spatial and temporal patterns of dengue incidence in Bhutan: a Bayesian analysis

**DOI:** 10.1080/22221751.2020.1775497

**Published:** 2020-06-15

**Authors:** Tsheten Tsheten, Archie C.A. Clements, Darren J. Gray, Sonam Wangchuk, Kinley Wangdi

**Affiliations:** a Department of Global Health, Research School of Population Health, Australian National University, Canberra, Australia; b Royal Centre for Disease Control, Ministry of Health, Thimphu, Bhutan; c Faculty of Health Sciences, Curtin University, Perth, Australia; d Telethon Kids Institute, Nedlands, Australia

**Keywords:** Dengue, temporal, spatial, Bayesian, Bhutan

## Abstract

Dengue is an important emerging vector-borne disease in Bhutan. This study aimed to quantify the spatial and temporal patterns of dengue and their relationship to environmental factors in dengue-affected areas at the sub-district level. A multivariate zero-inflated Poisson regression model was developed using a Bayesian framework with spatial and spatiotemporal random effects modelled using a conditional autoregressive prior structure. The posterior parameters were estimated using Bayesian Markov Chain Monte Carlo simulation with Gibbs sampling. A total of 708 dengue cases were notified through national surveillance between January 2016 and June 2019. Individuals aged ≤14 years were found to be 53% (95% CrI: 42%, 62%) less likely to have dengue infection than those aged >14 years. Dengue cases increased by 63% (95% CrI: 49%, 77%) for a 1°C increase in maximum temperature, and decreased by 48% (95% CrI: 25%, 64%) for a one-unit increase in normalized difference vegetation index (NDVI). There was significant residual spatial clustering after accounting for climate and environmental variables. The temporal trend was significantly higher than the national average in eastern sub-districts. The findings highlight the impact of climate and environmental variables on dengue transmission and suggests prioritizing high-risk areas for control strategies.

## Introduction

Dengue is a disease caused by arthropod-borne flaviviruses belonging to one or more of the four dengue virus serotypes (DENV1-4) [1]. An infection with one DENV serotype confers long-term immunity to that particular serotype, while a secondary infection with a different serotype (secondary heterotypic infection) can result in a severe form of the disease known as severe dengue or dengue haemorrhagic fever [2]. DENV-2 and DENV-3 are more commonly associated with hospitalization and greater mortality than the other two serotypes [3, 4].

Dengue transmission occurs through the bite of *Aedes* mosquitoes, namely, *Ae. aegypti* and *Ae. albopictus*. *Ae. aegypti* is most commonly found in tropical and sub-tropical regions, while *Ae. albopictus* has a larger geographical range due to its tolerance of colder temperatures [5]. *Ae. aegypti* typically breeds in man-made containers available in an urban environment, and rests and bites both indoors and outdoors [6]. *Ae. albopictus* is mostly an outdoor mosquito that breeds in natural containers such as banana trees, tree holes, coconut shells and cut bamboo [7]. Both species feed during the day time from morning until dusk, although night-time biting is sometimes observed in *Ae. albopictus* [8].

The global burden of dengue has increased over the last few decades from over 8.3 million cases in 1990 to more than 58.4 million cases in 2013 [9]. Recent research estimates that there are 390 million dengue infections worldwide and 21,000 deaths due to severe dengue every year [10]. Generally, dengue viruses are associated with only mild illness during inter-epidemic periods (silent transmission), however, major epidemics occur every 3–5 years [11]. This interannual periodicity occurs because, after large epidemics, subsequent large epidemics do not reoccur for some time as a result of long-term protection against the infecting serotype and transient cross-protection to other serotypes [12]. The population remains susceptible to future outbreak/s due to waning of this transient immunity and the low level of herd immunity. Low herd immunity occurs due to vector control activities, which protects the people from the bite of *Aedes* mosquitoes, thereby preventing people from acquiring natural immunity [13].

Climate plays a major role in the global and local spread of dengue infection [6, 14]. Epidemic transmission of dengue fever is strongly correlated with fluctuations in rainfall, temperature, relative humidity and vegetation types. Changes in these variables affect the population dynamics of mosquito vectors and the consequent risk of dengue transmission [14]. Increased precipitation during the monsoon season facilitates vector population growth by providing aquatic media for mosquitoes, although excessive rainfall can lead to flushing out of breeding sites and killing of mosquito larva [15, 16]. In some urban regions, stable larval habitats are provided by large domestic water tanks that are used to manage periodic water shortages [17]. Increased temperature due to global warming has been shown to enhance the transmission of dengue by favouring virus replication, proliferation and feeding behaviour of the vector [18]. Conversely, extreme hot weather may reduce transmission by increasing the mosquito mortality rate [19]. Increasing relative humidity is associated with increased oviposition, greater vector survival and larger numbers of infected mosquitoes [20]. Vegetation cover (as indicated by land surface greenness) influences the risk dengue transmission as vegetation provides resting or breeding sites for mosquitoes [21]. However, previous studies have reported inconsistent relationships between greenness indices and dengue incidence; some studies indicating a positive association [22], while others finding a negative association [23]. Choi et al. also showed that the relationship between dengue incidence and climatic variables varies by locality, suggesting that climate-based early warning should be established at the local scale [24]. Hence, understanding the local spatial and temporal dynamics of dengue, and recognizing the relationship between dengue incidence and climatic variables is essential to target surveillance and control efforts in a given area.

Various analytical approaches have been used to describe the spatio-temporal dynamics of dengue transmission using climate and environmental variables. Most of those studies have adopted a frequentist approach of analysis, where parameters are derived from the likelihood alone [20, 25]. Recently, Bayesian methods have been used to analyse the spatio-temporal distribution of dengue transmission. Bayesian methods have the flexibility of incorporating prior knowledge (including in relation to the spatial and temporal structure of the data), and to model spatial (and temporal) correlation simultaneously with the effect of covariates [26]. The Bayesian approach has the advantage of fully quantifying uncertainty in the estimates and the capability of dealing with small sample sizes [27]. Here, we used a Bayesian approach to analyse the spatio-temporal distribution of dengue in Bhutan to assist dengue control programmes to allocate limited resources to areas with high dengue transmission.

In Bhutan, dengue viruses have been continuously circulating for nearly two decades with the first report of dengue in 2004. The first documented outbreak of dengue was reported in the Phuentsholing sub-district in Chukha district, which shares a porous border with India [28]. By 2018, seven other neighbouring districts also reported dengue, either as an isolated case or as outbreaks. Past dengue outbreaks were associated with co-circulation of DENV-1–3, while DENV-4 is not yet detected in the country [28, 29].

Dengue is one of the important disease managed by the Vector-borne Disease Control Program (VDCP), of the Ministry of Health since 2003. The VDCP supports, coordinates and evaluates vector control activities in district hospitals and Basic Health Units (BHUs) [30]. All health centres in Bhutan follow the same guidelines and policies developed by the Ministry. Vector surveillance and control is the primary tool used to prevent and control dengue in Bhutan, through source reduction of mosquitoes, indoor residual spraying (IRS) of insecticide, thermal fogging, and health promotion activities. Separate to this, dengue disease surveillance is led by Royal Centre for Disease Control (RCDC), another division in the Ministry of Health. This system is designed to detect cases early and provide an efficient and timely response for control of the disease.

This study was undertaken to investigate the spatial and temporal patterns of dengue in Bhutan and quantify the role of local environmental factors in dengue fever transmission. This is the first study in the country to explore relationships between climate, and other environmental factors, and dengue incidence.

## Materials and methods

### Study setting

Bhutan is one of Asia's smallest nations, situated in the southern slopes of the eastern Himalayas bordering China in the North and India in the South, West and East. The country has a total area of 38,394 square kilometres and lies between 27° 30′ N and 90° 30′ E. Bhutan is administratively divided into 20 districts, which are further broken down into 205 sub-districts.

**Figure 1.**
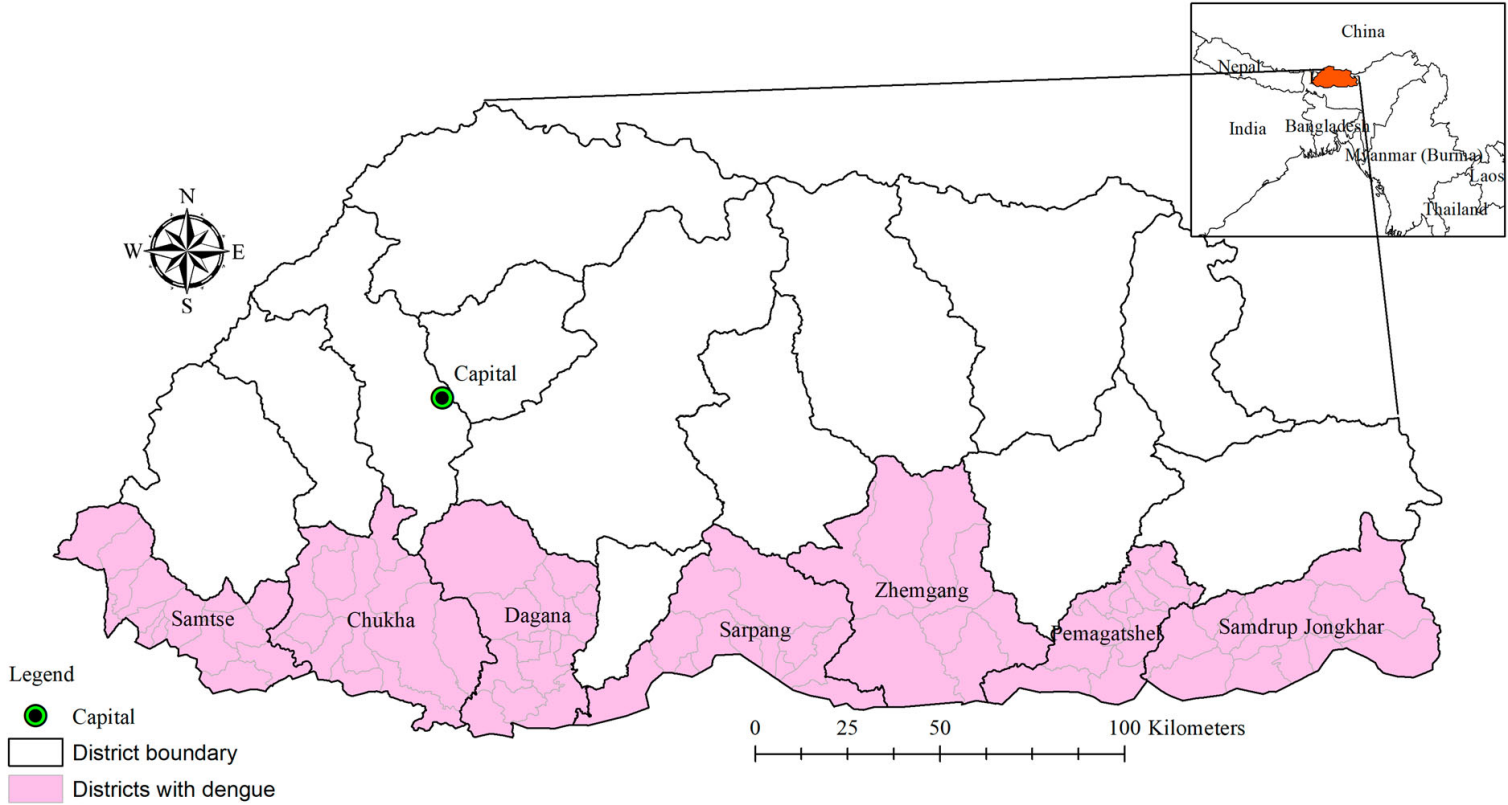
Administrative map of Bhutan and districts with documented dengue.

Seven districts where dengue transmission has been documented were included in the study. These districts include 82 sub-districts (Figure 1).

### Data sources

### Dengue cases

Weekly numbers of dengue cases were obtained from the National Early Warning, Alert, Response and Surveillance (NEWARS) database housed in the RCDC and aggregated at monthly intervals. NEWARS is an integrated online disease surveillance system that collates the notification of prioritized national notifiable diseases and syndromes, including dengue, from health centres across the country. The reporting can be done either through an online web-based system or a mobile-based short message service (SMS) to report cases from remote places with limited internet access. All dengue fever cases are required to be reported into NEWARS from all health centres every week. Dengue cases are defined as any patients presenting fever with any two of the following symptoms: headache, retro-orbital pain, myalgia, arthralgia, rash, positive tourniquet test and any warning signs of severe dengue (abdominal pain, persistent vomiting, mucosal bleeding, fluid accumulation, liver enlargement and increasing haematocrit value) [11]. Cases included in the study were all confirmed by rapid diagnostic tests and clinical-epidemiological criteria (those living in a dengue-endemic area or those with a recent history of travel to a dengue-endemic area and developing the symptoms described above). Clinicians and other health professionals are trained in the identification and reporting of dengue. When reporting to NEWARS, all cases are classified into following age groups: 1–29 days, 1–11 months, 1–4 years, 5–9 years, 10–14 years, 15–19 years, 20–24 years, 25–49 years, 50–64 years and ≥65 years. In our study, cases were categorized into two groups: ≤14 years and >14 years.

### Environmental and climate variables

Ten-day composites of 250-m spatial resolution normalized difference vegetation index (NDVI) data for the study period were obtained from the U.S. Geological Survey's (USGS) Earth Resources Observation and Science (EROS) Moderate Resolution Imaging Spectroradiometer (eMODIS) (https://earthexplorer.usgs.gov/) [31]. NDVI indicates vegetation greenness and is often used as a proxy for mosquito-favoured habitats [22]. Prior to analysis, three 10-day composited raster images were used to obtain mean NDVI values by monthly windows for the study area. The spatial analyst tool (zonal statistics) in the geographical information system (GIS) software ArcGIS version 10.5 (ESRI, Redlands, CA) was used to extract the means for each sub-district [32].

Monthly data on climatic variables (rainfall, relative humidity, minimum and maximum temperature) were obtained from the National Centre for Hydrology and Meteorology of the Royal Government of Bhutan. The Centre collects data on these climatic variables from weather stations located in each district. Average monthly data of the district were calculated for each month of the study period from the daily records. District climatic variables were interpolated to the sub-districts within each district because sub-districts climatic records are unavailable. Population of the sub-districts was obtained from the National Statistical Bureau, Royal Government of Bhutan [33].

### Crude standardized morbidity ratios

Raw standardized morbidity ratios (SMR) were calculated for each sub-district using the following formula:

$$Y_{i}=\frac{O_{i}}{E_{i}}$$

where *Y_i_* is the SMR for the *i*th sub-district, *O_i_* is the observed number of dengue cases in the *i^t^*^h^ sub-district, and *E_i_* is the expected number of dengue cases in the *i*^th^ sub-district. The expected number of dengue cases was calculated by multiplying the national incidence of dengue by the population of the sub-district during the study period.

### Exploration of temporal trends of dengue

The monthly mean number of dengue cases was calculated for the full-time series (January 2016 to June 2019). The time series was then decomposed into three temporal components using locally-weighted regression or loess: seasonality, trend and residual variability [34]. The data, the trend component, the seasonal component and the residual component are denoted by *Y_t_, T_t_, S_t_* and *R_t_*, respectively, for *t*=1 to N. The formula for the loess model is:

$$Y_{t}\;=\;T_{t}+\;S_{t}+\;R_{t}$$

The function *stl* and parameter setting *periodic* were used to decompose the time series data and extract all the above parameters using R studio [35]. In the final model, a logarithmic transformation was used to assess the significance level of the trend.

### Spatio-temporal models

An initial univariate Poisson regression analysis was performed to select covariates, with number of dengue cases as the dependent variable and NDVI and climatic variables with 0, 1, 2 and 3 months lag as independent variables. Lags for each variable with the lowest Akaike Information Criterion (AIC) [36] and a significant Incidence Rate Ratio (IRR) with a *p*-value <0.05 were selected. IRR was calculated by exponentiating the estimated regression coefficients from the Zero-inflated Poisson analysis output [37]. The collinearity of the selected covariates (temperature, humidity and NDVI) was tested using variance inflation factor (VIF) diagnostic tool [38]. Covariates with a VIF >4.0 were considered to be collinear and were removed from the final model. In this study, the final model contained rainfall and relative humidity lagged at 2 months, maximum temperature without lag, and NDVI lagged at 1 month (Supplementary Table).

The number of cells containing zero counts in our dataset was 6,775 (98%). So, the analysis was done to choose the best fitting model using AIC and Bayesian Information Criteria (BIC) [36]. Zero-inflated Poisson (ZIP) regression showed better fit over standard Poisson regression with lower AIC and BIC. A significant difference was observed between the two models as demonstrated by the Vuong test (Supplementary Table). ZIP regression models were constructed in a Bayesian framework. Model I contained independent variables (age, rainfall, maximum temperature, relative humidity and NDVI), unstructured and spatiotemporal random effects for the sub-districts (i.e. no spatial autocorrelation was assumed in the relative risk of dengue, but was assumed in the values of the district-level trends); in Model II, the unstructured random effects were replaced with spatially structured random effects but otherwise contained all of the same model components (described below); and the final Model III included all of the components of Model I and Model II, that is: unstructured, structured and spatiotemporal random effects.

In the last model, dengue incidence in sub-district *i* (1, … ,82), month *j* was modelled using ZIP regression as follows:

$$P\;(Y_{\mathrm{ij}}=\;y_{\mathrm{ij}})=\;\{\begin{array}{c} \omega +1(1-\omega )e^{-\mu },\;\;\;\;\;\;\;y_{\mathrm{ij}}\;=0\\ (1-\omega )e^{-\mu }\mu_{\mathrm{ij}}^{\mathrm{yij}}/y_{\mathrm{ij}},\;\;\;\;y_{\mathrm{ij}}\;>0\;\; \end{array}$$

$$Y_{\mathrm{ij}}∼\mathrm{Poisson}(\mu_{\mathrm{ij}})$$

$$\log(\mu_{\mathrm{ij}})=\log(E_{i})+\theta_{\mathrm{ij}}$$

$$\theta_{\mathrm{ij}}=\alpha +\beta_{1}\mathrm{Age}+\beta_{2}\mathrm{Rainfall}+\beta_{3}T\max+\beta_{4}\mathrm{Humid}+\beta_{5}\mathrm{NDVI}+u_{i}+v_{\mathrm{ij}}+w_{\mathrm{ij}}$$

where *y_ij_* is the number of dengue cases in *i*=1, … ., 82 sub-districts and month *j*; ω indicates the probability of being an excess zero, $\mu_{\mathrm{ij}}$ is the mean number of cases, *E_i_* is the expected number of cases (acting as an offset to control for population size); *θ_ij_* is the log relative risk of dengue; *α* is an intercept; *β_1_*, *β_2,_ β_3,_ β_4,_* and *β_5_*_,_ are the coefficients for age (over 15 years as a reference and assigned 0), rainfall lagged at 2 months, maximum temperature (^⁰^C) without lag, relative humidity lagged at 2 months and NDVI lagged at 1 month respectively; *u_i_* are unstructured random effects with a zero mean and variance *σ_u_^2^*, *v_i_* are spatially structured random effect with a zero mean and variance σ_v_^2^ and w*_ij_* is the spatiotemporal random effect with a mean of zero and variance of σ_w_^2^.

A conditional autoregressive (CAR) prior structure was used to model the spatially structured and spatiotemporal random effects. Spatial relationships between sub-districts were structured using an adjacency weight matrix, with a weight of “1” for contiguous and “0” for non-contiguous sub-districts. We specified a flat prior distribution for the intercept and a normal prior distribution for the random effects (mean of “0” and precision, inverse of variance, set at 0.0001). The priors for the precision of unstructured, structured and spatiotemporal random effects (inverses of variances shown above), were specified using non-informative gamma distributions with shape and scale parameters equal to 0.001.

An initial burn-in of 10,000 iterations was run, which was discarded. Model fitting was carried out using three separate chains starting at different initial values. Convergence was assessed by visualization of posterior density and history plots, and by computing the Gelman and Rubin diagnostic. After burn-in, subsequent blocks of 10,000 iterations were run and inspected for convergence. Thinning by a factor of 20 was specified after 40,000 iterations due to poor mixing of the chains and within-chain autocorrelation. Convergence occurred at approximately 100,000 iterations for each model. The posterior distributions of each model parameter were stored following convergence and summarized for the analysis using posterior mean and 95% credible intervals (95% CrI). The model with the lowest deviance information criterion (DIC) was selected as the best-fit model.

In all the analyses, an α-level of 0.05 was adopted to indicate statistical significance (as indicated by 95% CrI for relative risks that excluded 1). Time series analysis with cross-correlations and univariate regression analysis was performed using Stata statistical software (version 15.1; Stata Corp, College Station, Texas, USA) [39]. The Bayesian models were developed using the WinBugs statistical software version 1.4.3 (Medical Research Council, Cambridge, UK) [40]. Chloropleth maps of SMR and the random effects were produced using ArcGIS version 10.5 with shapefiles [32].

Ethical clearance for this study was approved by the Research Ethics Board of Health (REBH), Ministry of Health, Bhutan (REBH/Approval/2019/040) and Australian National University (ANU), Australia (2019/816).

## Results

### Descriptive statistics

**Figure 2.**
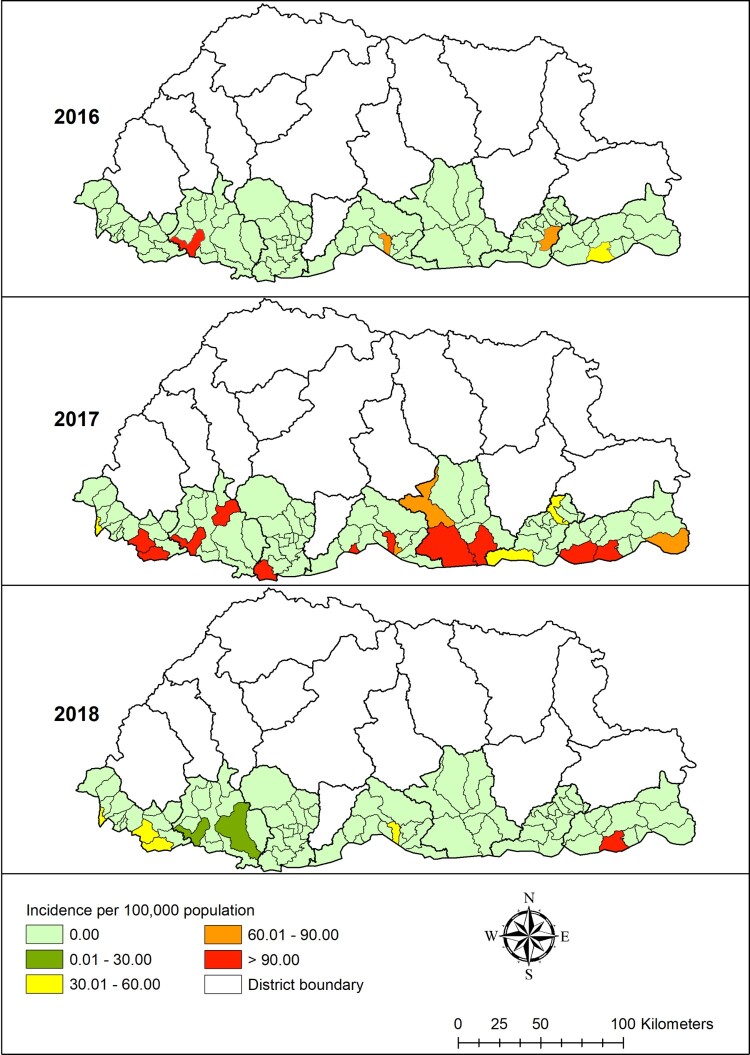
Dengue incidence rates by sub-districts, Bhutan, 2016–2018

A total of 708 dengue cases were notified to NEWARS from January 2016 to June 2019. The age-specific incidence rate of dengue was 155 per 100,000 inhabitants aged ≤14 years, 263 per 100,000 inhabitants aged >14 years. Chukha district reported the highest number of dengue cases followed by Samdrup Jongkhar and Samtse, with means of 0.69 (range: 0–72), 0.43 (range:0–76) and 0.18 (range: 0–40) cases per month respectively (Table 1). The distribution of dengue varied greatly between sub-districts, with incidence rate ranging from zero to as high as >90 per 100,000 inhabitants each year during the study period (Figure 2).

**Table 1.** Distribution of monthly means of dengue fever cases and climate and environmental variables in Bhutan, January 2016–June 2019.

Mean (Minimum–Maximum)						
Districts	No. of sub-districts	Cases	Rainfall (mm)	Maximum temperature °C	Relative humidity (%)	NDVI
Chukha	11	0.69 (0.00–72.00)	11.52 (0.00–50.23)	29.29 (23.61–32.73)	71.30 (31.56–88.67)	0.46 (−0.01–0.98)
Dagana	14	0.01(0.00–3.00)	2.55 (0.00–15.37)	21.24 (11.32–26.50)	66.71 (44.43–92.80)	0.53 (0.01–0.95)
Pemagatshel	11	0.01(0.00–1.00)	3.52 (0.00–23.72)	21.81 (14.48–26.56)	82.60 (73.67–90.25)	0.61 (0.02–0.91)
Samdrup Jongkhar	11	0.43 (0.00–76.00)	7.79 (0.00–30.02)	25.41 (20.11–29.98)	74.38 (47.74–93.51)	0.47 (−0.01–0.88)
Samtse	15	0.18 (0.00–40.00)	12.62 (0.00–47.18)	28.47 (21.40–32.08)	71.00 (47.22–90.54)	0.41 (−0.06–0.98)
Sarpang	12	0.12 (0.00–13.00)	12.58 (0.00–79.73)	27.41 (21.60–33.60)	80.20 (60.25–93.03)	0.48 (−0.01–0.97)
Zhemgang	8	0.02 (0.00–2.00)	3.19 (0.00–19.01)	20.29 (12.51–26.84)	73.88 (54.74–89.64)	0.56 (0.02–0.92)
Overall	82	0.21(0.00–76.00)	7.95 (0–79.73)	25.08 (11.32–33.60)	73.96 (31.56–93.51)	0.51 (−0.06–0.98)

**Figure 3.**
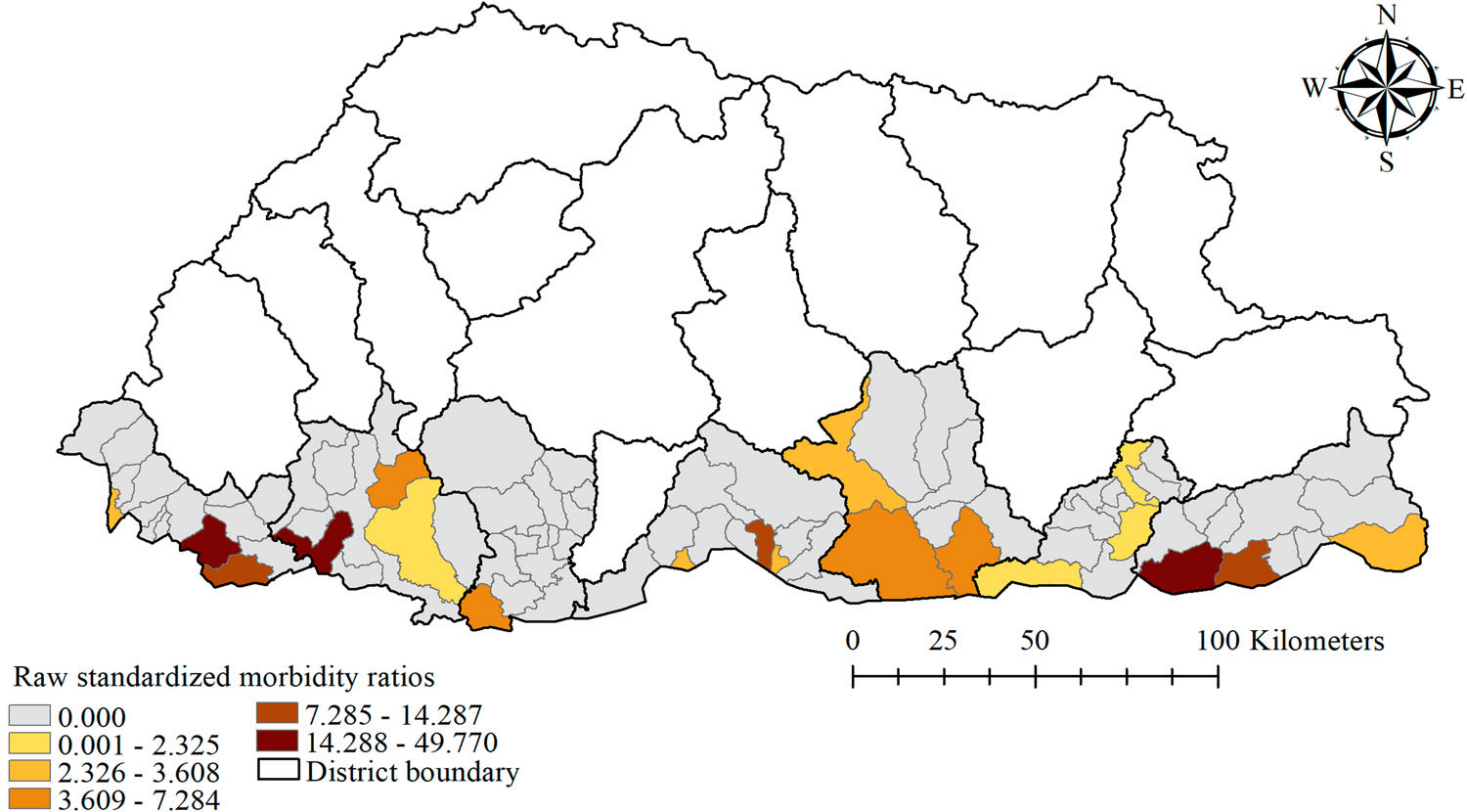
Raw standardized morbidity ratio of dengue by sub-districts in Bhutan, January 2016–June 2019

The monthly averages of rainfall, maximum temperature, relative humidity and NDVI of the study districts were 7.95 mm, 25.08°C, 73.96% and 0.51 respectively. The mean maximum temperature was highest in Chukha and Samtse districts (29.29°C and 28.47°C respectively), while these districts had the lowest NDVI values (0.46 and 0.41 respectively) (Table 1). There was wide spatial variation of SMR for dengue during the study period. The general pattern showed high SMR along the sub-districts located in the southern belt that shares a common boundary with India. Sub-districts with high SMR values (above 14.28) were Phuentsholing, Samtse and Dewathang (Figure 3).

### Time series decomposition

**Figure 4.**
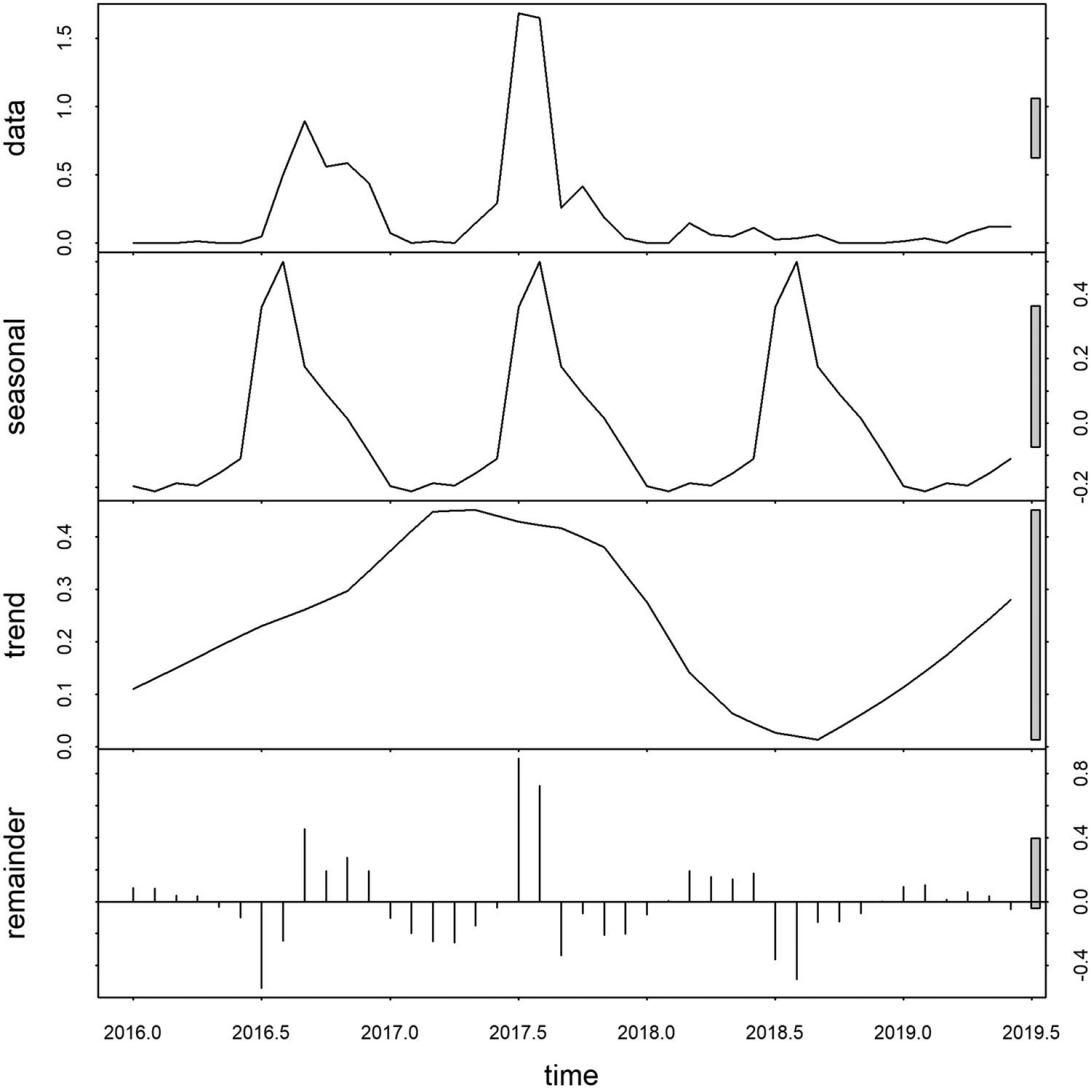
Temporal decomposition of numbers of dengue cases of Bhutan, January 2016–June 2019.

The raw data exhibited a seasonal pattern with an apparent increase in 2016 and 2017. The seasonal decomposition plot demonstrated strong seasonality with a peak occurring between July and September each year. The inter-annual pattern showed a large peak in 2017 and an apparently increasing trend in 2019. The large grey bar in the third panel shows that the variation in the trend component is small as compared to variation in the data and seasonality component. The residuals in the fourth panel showed slightly elevated variability in the earlier years (2016–2017) and less apparent variability in the subsequent years (Figure 4).

### Spatio-temporal model

**Figure 5.**
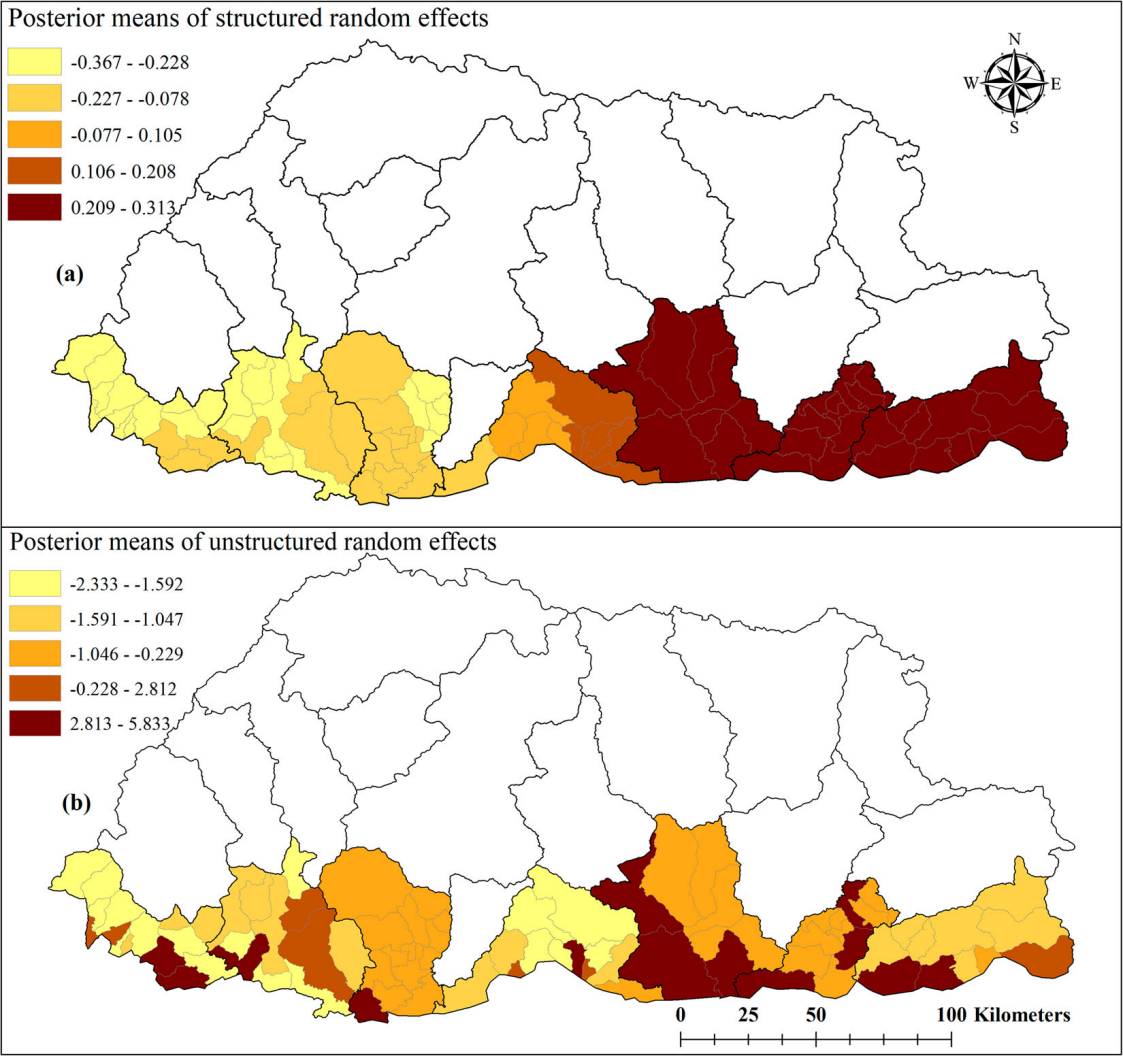
Spatial distribution of posterior means of structured (a) and unstructured random effects (b) in Bhutan, January 2016–June 2019 based on a Bayesian spatiotemporal model.

We chose the mixed model containing the unstructured, structured and spatiotemporal random effects (Model III). Whilst this model didn't have the lowest DIC, it had a DIC value nearly as low as the unstructured model (Model I) and provided more information on the patterns of dengue risk. Individuals aged ≤ 14 years were found to be 53% (95% CrI: 42%, 62%) less likely to have dengue infection than those aged > 14 years. Dengue cases increased by 63% (95% CrI: 49%, 77%) for a 1°C increase in maximum temperature; and decreased by 48% (95% CrI: 25%, 64%) for a one unit increase in NDVI (Table 2). Mapping of the spatially structured random effect (*v_i_*) showed higher mean dengue risk in Pemagatshel, Samdrup Jongkhar and some part of Zhemgang district (Figure 5(a)). The unstructured random effect (*u_i_*) unsurprisingly revealed a random distribution of posterior means (Figure 5(b)).

**Table 2.** Regression coefficients, relative risk and 95% credible interval from Bayesian spatial and non-spatial models of dengue cases in Bhutan, January 2016-June 2019.

Model/Variable	Coeff, posterior mean (95% CrI)	RR, posterior mean (95% CrI)
**Model I (Unstructured)**		
α (Intercept)^a^	−4.936 (−7.052, −3.481)	
Age (Above 14 years as base)	−0.752 (−969, −0.539)	0.471 (0.379, 0.582)
Mean monthly trend	1.023 (0.764, 1.325)	2.782 (2.146, 3.762)
Rainfall lagged 2 months (mm)	0.044 (−0.043, 0.132)	1.036 (0.965, 1.113)
Relative humidity lagged 2 months (%)	0.053 (−0.144, 0.252)	1.004 (0.988, 1.021)
Maximum temperature without lag (°C)	2.188 (1.824, 2.564)	1.619 (1.495, 1.759)
NDVI lagged 1 month	−0.666 (−1.036, −0.305)	0.514 (0.355, 0.737)
Probability of extra zero	0.702 (0.641, 0.758)	2.017 (1.898, 2.133)
Heterogeneity^a^		
Unstructured	0.112 (0.043, 0.221)	
Structured (trend)	23.88 (1.101, 152.600)	
DIC	1421.200	
**Model II (Structured)**		
α (Intercept)^a^	−4.699 (−6.413, −3.368)	
Age (Above 14 years as base)	−0.752 (−1.208, −0.319)	0.472 (0.298, 0.726)
Mean monthly trend	1.041 (0.541, 1.546)	2.832 (1.717, 4.693)
Rainfall lagged 2 months (mm)	0.046 (−0.134, 0.224)	1.038 (0.897, 1.199)
Relative humidity lagged 2 months (%)	0.045 (−0.351, 0.439)	1.004 (0.972, 1.036)
Maximum temperature without lag (°C)	2.254 (1.270, 3.345)	1.644 (1.323, 2.091)
NDVI lagged 1 month	−0.636 (−614, 0.283)	0.529 (0.199, 1.328)
Probability of extra zero	0.704 (0.629, 0.770)	2.022 (1.878, 2.159)
Heterogeneity^a^		
Structured (spatial)	0.044 (0.735, 39.700)	
Structured (trend)	7.048 (0.018, 0.086)	
DIC	1529.61	
**Model III (Mixed)**		
α (Intercept)^a^	−4.779 (−6.764, −3.468)	
Age (Above 14 years as base)	−0.753 (−0.972, −0.540)	0.471 (0.378, 0.582)
Mean monthly trend	1.023 (0.758, 1.327)	2.782 (2.133, 3.769)
Rainfall lagged 2 months (mm)	0.045 (−0.043, 0.133)	1.037 (0.966, 1.114)
Relative humidity lagged 2 months (%)	0.050 (−0.151, 0.249)	1.004 (0.988, 1.020)
Maximum temperature without lag (°C)	2.199 (1.818, 2.596)	1.625 (1.493, 1.772)
NDVI lagged 1 month	−0.663 (−1.040, −0.290)	0.515 (0.353, 0.748)
Probability of extra zero	0.703 (0.642, 0.759)	2.019 (1.900, 2.135)
Heterogeneity^a^		
Unstructured	0.126 (0.047, 0.259)	
Structured (spatial)	275.700 (0.593, 1843)	
Structured (trend)	38.740 (1.142, 349.500)	
DIC	1423.63	

Abbreviations: coeff: coefficients; CrI: credible interval; RR-relative risk; DIC-deviance information criterion.

^a^Coefficient.

**Figure 6.**
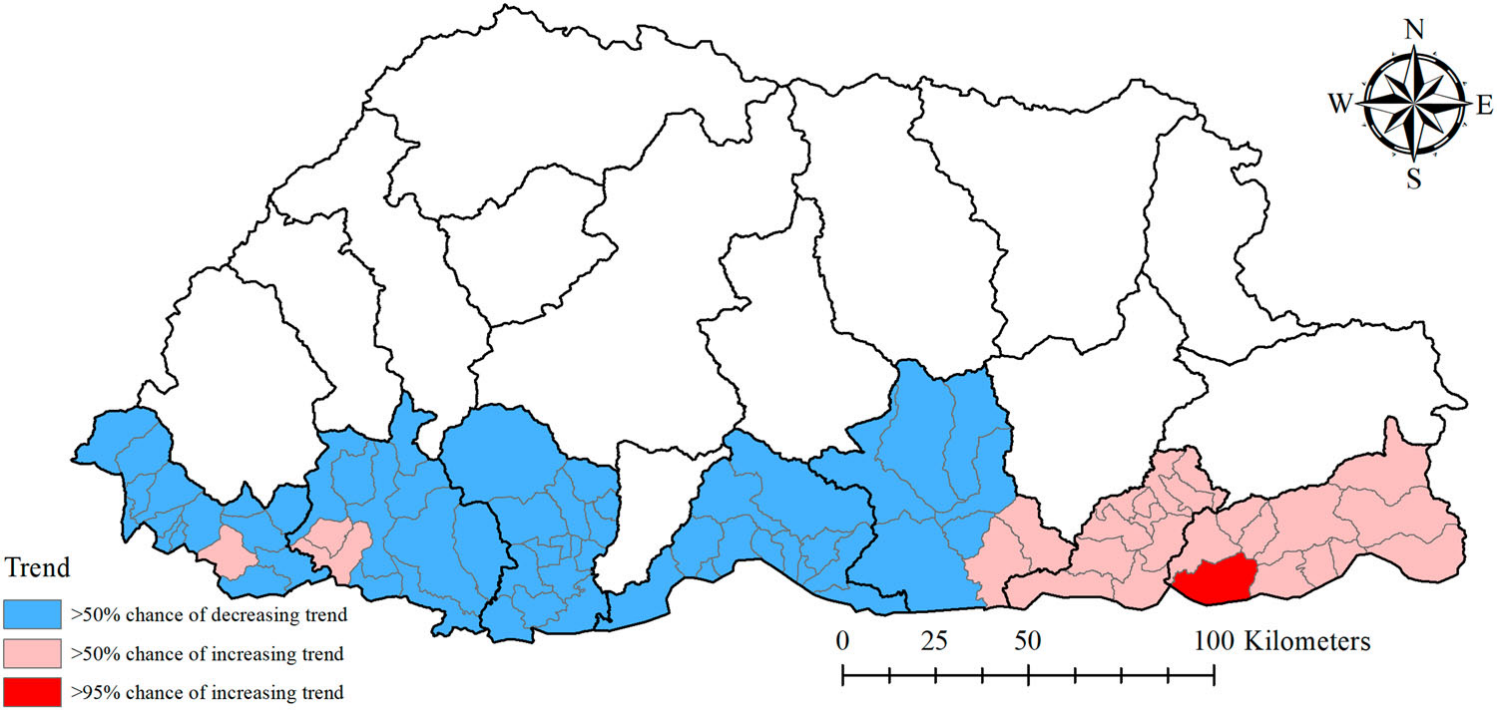
Trend analysis of dengue incidence in Bhutan, January 2016–June 2019, based on the spatiotemporal random effects of a Bayesian model.

Dewathang sub-district located in the eastern part of the region showed >95% probability of a higher than national average trend. There was >50% probability of higher than national average trend in 26 sub-districts, that are mostly located along the eastern region, indicating a higher rate of increase in the incidence of dengue in this part of the country (Figure 6).

## Discussion

The analysis demonstrated that the risk of dengue infection in those aged >14 years was significantly higher than in those aged ≤14 years. A similar increased in the risk of older people was observed in other countries of the WHO-South-East Asia Region (SEAR) such as Indonesia [41], Thailand [42] and Nepal [43]. This may be because adult populations are engaged more in outdoor activities that render them vulnerable to dengue mosquito bites [44]. In addition, dengue infection in adults mostly results in symptomatic cases as compared to children, in whom up to 95% of infections had been reported to mild or sub-clinical [45]. Paediatric patients are less likely to manifest dengue-like illness including warning signs as compared to older patients. In a retrospective study conducted by Bryne et al. (2017), fever was absent in 25% of the children below 15 years of age, that were laboratory confirmed for dengue [46]. As a result, diagnosis of dengue among those children are likely to be overlooked and not reported through the surveillance system.

A clear seasonal pattern was observed during the study period. Time series analysis showed dengue as a highly seasonal disease with peaks observed in the monsoon, which occurs from June to August each year (Supplementary figure). The monsoon is correlated with an extended period of rainy and wet days, and higher temperatures, which are favourable for mosquito breeding and population expansion [15, 16]. The finding is consistent with studies conducted in Indonesia [22], Timor-Leste [47] and Sri Lanka [48], which demonstrated an increasing number of cases during the monsoon and hottest season.

Time-series analysis also demonstrated large interannual variability with dramatic increase of dengue incidence in 2017. Such an increase can be explained by the geographical expansion of dengue to new geographical regions. From 2017 onwards, dengue cases were reported from Samtse, Samdrup Jongkhar, Pemagatshel, Sarpang, Dagana, Samtse and Zhemgang districts in addition to Chukha district. These districts are located along the international border with India. One plausible reason is importation of dengue from India across a highly porous border. Dengue cases in Bhutan increased following an upsurge of dengue cases in India along the international border [49]. The people living along the border areas in these districts freely cross to India during the day time and possibly got exposed to dengue. Other studies also found a higher incidence of dengue in districts bordering India [50].

From maps of the model posterior outputs, it is clear that cases in the newly affected areas are increasing with the highest risk observed in a recently dengue infected sub-districts. In particular, Dewathang sub-district had the highest upward trend as compared to other sub-districts. Plausible reasons could include unplanned urbanization and development in the area in addition to favourable climate for dengue because of its lower altitude. Additionally, NK Darranga is one of the fastest-growing Indian towns along the Indo-Bhutan border and is located adjacent to Samdrup Jongkhar Town in Bhutan, with the towns being a walkable distance from each other. Individuals are allowed to move freely for cross-border shopping and employment opportunities. The developmental activities and the frequent migration of people across the border could have facilitated the transmission of dengue fever [43, 51]. All this evidence suggests that surveillance should be strengthened in the country with focus on point of entry in collaboration with Indian counterparts.

Maximum temperature was strongly associated with dengue incidence in our model. A temperature of more than 16°C is required for many tropical species of mosquitoes to complete their life cycles [52]. The threshold survival temperature for dengue virus is estimated at 11.9°C, while the optimum survival temperature for *Aedes* mosquitoes ranges from 22 to 26°C, and vector cease to feed when temperature falls below 17°C [20, 53]. Optimum temperatures prolong the life expectancy of mosquitoes and facilitates their dispersal across geographical regions [54]. Higher temperature also reduces the extrinsic incubation period (EIP) of DENV in *Aedes* mosquitoes and promotes viral transmission [20, 47, 55]. The shortening of the EIP can increase the proportion of infectious *Aedes* mosquitoes before they die. Further, warmer temperature speeds up the gonotropic cycle and makes *Aedes* mosquitoes more aggressive, which increases the biting rate and frequency of dengue virus transmission [56].

By contrast, NDVI was negatively associated with dengue incidence. The negative association between NDVI and dengue incidence was also reported in other studies [23, 57, 58, 59]. Lower NDVI is seen in areas with a higher population density (including urban and peri-urban areas), which tend to be areas that are favourable to *Aedes* mosquitoes [59]. Both *Ae. aegypti* and *Ae. albopictus* are detected in Chukha, Samtse and Samdrup Jongkhar districts, while other districts included in the study are known to have only *Ae. albopictus* [29].

This study is subject to a number of limitations. First, the time series data for this study was three years and six months in duration only, due to unavailability of earlier data. This short study duration might have affected the findings of our spatio-temporal model. Second, the passive surveillance data are subjected to under-reporting because sub-clinical dengue cases not seeking medical attention were unlikely to have been picked up by the surveillance system. Third, cases reported from each sub-districts or districts were not known of its source of infection, whether locally acquired within the sub-district or imported from other dengue endemic places. However, the number of cases presented here are based on the notification of data by health centres in each sub-districts or districts as per the existing surveillance system. Fourthly, district climatic factors were interpolated to the sub-district. This could have led to cofounding of the results. But we believe that the climatic factors within a district to be generally homogenous due to small area of the districts. Finally, observed spatial and temporal variability might have been confounded by other factors such as socio-economic and ecological factors which are not included in the study. Future studies should include these variables to assess the true temporal relationship.

Overall, the number of notified dengue cases showed an increasing trend with strong seasonality observed during the monsoon season. The highest risk of dengue infections was observed in people aged >14 years. A significant association between dengue incidence and climatic factors indicates a crucial role for climatic conditions in driving the intensity of dengue virus transmission in the country. This finding could support the development of decision support tools that integrate climate data to establish climate-based early warning systems to provide informed decisions in implementing dengue control activities. High risk areas found in this study may be targeted for prioritizing allocation of resources to activities such as surveillance and vector control.

## References

[1] Huang Y-JS, Higgs S, Horne KM, et al. Flavivirus-mosquito interactions. Viruses. 2014;6(11):4703–4730. 25421894 10.3390/v6114703 PMC4246245

[2] Alvarez M, Rodriguez-Roche R, Bernardo L, et al. Dengue hemorrhagic fever caused by sequential dengue 1-3 virus infections over a long time interval: Havana epidemic, 2001-2002. Am J Trop Med Hyg. 2006;75(6):1113–1117. 17172378

[3] Wunderlich J, Acuna-Soto R, Alonso WJ. Dengue hospitalisations in Brazil: annual wave from West to East and recent increase among children. Epidemiol Infect. 2018;146(2):236–245. 29235427 10.1017/S0950268817002801 PMC9148759

[4] Diaz-Quijano FA, Waldman EA. Factors associated with dengue mortality in Latin America and the Caribbean, 1995-2009: an ecological study. Am J Trop Med Hyg. 2012;86(2):328–334. 22302870 10.4269/ajtmh.2012.11-0074 PMC3269288

[5] Brady OJ, Johansson MA, Guerra CA, et al. Modelling adult *Aedes aegypti* and *Aedes albopictus* survival at different temperatures in laboratory and field settings. Parasit Vectors. 2013;6(1):351. 24330720 10.1186/1756-3305-6-351 PMC3867219

[6] Ebi KL, Nealon J. Dengue in a changing climate. Environ Res. 2016;151:115–123. 27475051 10.1016/j.envres.2016.07.026

[7] Naish S, Dale P, Mackenzie JS, et al. Spatial and temporal patterns of locally-acquired dengue transmission in northern Queensland, Australia, 1993-2012. PLoS One. 2014;9(4):e92524. 24691549 10.1371/journal.pone.0092524 PMC3972162

[8] Higa Y. Dengue vectors and their spatial distribution. Trop Med Health. 2011;39(4 Suppl):17–27. 10.2149/tmh.2011-S04 PMC3317606 22500133

[9] Stanaway JD, Shepard DS, Undurraga EA, et al. The global burden of dengue: an analysis from the global burden of disease study 2013. Lancet Infect Dis. 2016;16(6):712–723. 26874619 10.1016/S1473-3099(16)00026-8 PMC5012511

[10] Bhatt S, Gething PW, Brady OJ, et al. The global distribution and burden of dengue. Nature. 2013;496:504. 23563266 10.1038/nature12060 PMC3651993

[11] World Health Organization. Dengue guidelines for diagnosis, treatment, prevention and control: New Edition. Geneva: World Health Organization; 2009. 23762963

[12] Ramadona AL, Lazuardi L, Hii YL, et al. Prediction of dengue outbreaks based on disease surveillance and Meteorological data. PLoS One. 2016;11(3):e0152688. 27031524 10.1371/journal.pone.0152688 PMC4816319

[13] Ooi E-E, Goh K-T, Gubler DJ. Dengue prevention and 35 years of vector control in Singapore. Emerg Infect Dis. 2006;12(6):887–893. 16707042 10.3201/10.3201/eid1206.051210 PMC3373041

[14] Murray NE, Quam MB, Wilder-Smith A. Epidemiology of dengue: past, present and future prospects. Clin Epidemiol. 2013;5:299–309. 23990732 10.2147/CLEP.S34440 PMC3753061

[15] Withanage GP, Viswakula SD, Nilmini Silva Gunawardena YI, et al. A forecasting model for dengue incidence in the district of Gampaha, Sri Lanka. Parasit Vectors. 2018;11(1):262. 29690906 10.1186/s13071-018-2828-2 PMC5916713

[16] Das M, Gopalakrishnan R, Kumar D, et al. Spatiotemporal distribution of dengue vectors & identification of high risk zones in district Sonitpur, Assam, India. Indian J Med Res. 2014;140(2):278–284. 25297362 PMC4216503

[17] Beebe NW, Cooper RD, Mottram P, et al. Australia’s dengue risk driven by human adaptation to climate change. PLoS Negl Trop Dis. 2009;3(5):e429. 19415109 10.1371/journal.pntd.0000429 PMC2671609

[18] Banu S, Hu W, Hurst C, et al. Dengue transmission in the Asia-Pacific region: impact of climate change and socio-environmental factors. Trop Med Int Health. 2011;16(5):598–607. 21320241 10.1111/j.1365-3156.2011.02734.x

[19] Hii YL, Rocklöv J, Ng N, et al. Climate variability and increase in intensity and magnitude of dengue incidence in Singapore. Glob Health Action. 2009: 2. doi:10.3402/gha.v2i0.2036. PMC2799326 20052380

[20] Ramachandran VG, Roy P, Das S, et al. Empirical model for estimating dengue incidence using temperature, rainfall, and relative humidity: a 19-year retrospective analysis in East Delhi. Epidemiol Health. 2016;38:e2016052. 27899025 10.4178/epih.e2016052 PMC5309726

[21] Martinez-Bello DA, Lopez-Quilez A, Torres Prieto A. Relative risk estimation of dengue disease at small spatial scale. Int J Health Geogr. 2017;16(1):31. 28810908 10.1186/s12942-017-0104-x PMC5558735

[22] Astuti EP, Dhewantara PW, Prasetyowati H, et al. Paediatric dengue infection in Cirebon, Indonesia: a temporal and spatial analysis of notified dengue incidence to inform surveillance. Parasit Vectors. 2019;12(1):186. 31036062 10.1186/s13071-019-3446-3 PMC6489314

[23] Huang C-C, Tam TYT, Chern Y-R, et al. Spatial clustering of dengue fever incidence and Its association with Surrounding greenness. Int J Environ Res Public Health. 2018;15(9):1869. 30158475 10.3390/ijerph15091869 PMC6163306

[24] Choi Y, Tang CS, McIver L, et al. Effects of weather factors on dengue fever incidence and implications for interventions in Cambodia. BMC Public Health. 2016;16:241. 26955944 10.1186/s12889-016-2923-2 PMC4784273

[25] Ling CY, Gruebner O, Kramer A, et al. Spatio-temporal patterns of dengue in Malaysia: combining address and sub-district level. Geospat Health. 2014;9(1):131–140. 25545931 10.4081/gh.2014.11

[26] Hu W, Clements A, Williams G, et al. Spatial patterns and socioecological drivers of dengue fever transmission in Queensland, Australia. Environ Health Perspect. 2012;120(2):260–266. 22015625 10.1289/ehp.1003270 PMC3279430

[27] van de Schoot R, Kaplan D, Denissen J, et al. A gentle introduction to Bayesian analysis: applications to developmental research. Child Dev. 2014;85(3):842–860. 24116396 10.1111/cdev.12169 PMC4158865

[28] Dorji T, Yoon IK, Holmes EC, et al. Diversity and origin of dengue virus serotypes 1, 2, and 3, Bhutan. Emerg Infect Dis. 2009;15(10):1630–1632. 19861059 10.3201/eid1510.090123 PMC2866390

[29] Zangmo S, Klungthong C, Chinnawirotpisan P, et al. Epidemiological and Molecular Characterization of dengue virus circulating in Bhutan, 2013-2014. PLoS Negl Trop Dis. 2015;9(8):e0004010. 26295474 10.1371/journal.pntd.0004010 PMC4546418

[30] Ministry of Health. Annual health bulletin. Thimphu: Health Management and Information System; 2004.

[31] Jenkerson CB, Maiersperger W, et al. eMODIS: A user-friendly data source: U.S. Geological Survey Open-File Report 2010–1055, 10 p. 2010.

[32] ESRI. ArcGIS 10.5 for desktop. Environmental System. Redlands (CA): Research Institute (ESRI).

[33] National Statistical Bureau. Dzongkhag population projections 2017–2027; 2017.

[34] Cleveland RB, Cleveland WS, McRae JE, et al. STL: a seasonal-trend decomposition. J Off Stat. 1990;6(1):3–73.

[35] R Core Team. R: A Language and environment for statistical computing: R Foundation for statistical computing. Vienna: R Core Team; 2019.

[36] Burnham KP, Anderson DR. Multimodel inference: understanding AIC and BIC in model selection. Sociol Methods Res. 2004;33(2):261–304.

[37] Parodi S, Bottarelli E. Poisson regression model in epidemiology—an introduction. Ann Fac Medic Vet di Parma. 2006;26:25–44.

[38] O’brien RM. A caution regarding rules of thumb for variance inflation factors. Qual Quant. 2007;41(5):673–690.

[39] StataCorp. Stata statistical software: Release 15. College Station (TX): StataCorp LLC.

[40] Spiegelhalter DJ, Thomas A, Best NG. WinBUGS Version 1.2 User Manual. Cambridge: MRC Biostatistics Unit.

[41] Karyanti MR, Uiterwaal CS, Kusriastuti R, et al. The changing incidence of dengue haemorrhagic fever in Indonesia: a 45-year registry-based analysis. BMC Infect Dis. 2014;14:412. 25064368 10.1186/1471-2334-14-412 PMC4122763

[42] Wichmann O, Hongsiriwon S, Bowonwatanuwong C, et al. Risk factors and clinical features associated with severe dengue infection in adults and children during the 2001 epidemic in Chonburi, Thailand. Trop Med Int Health. 2004;9(9):1022–1029. 15361117 10.1111/j.1365-3156.2004.01295.x

[43] Khetan RP, Stein DA, Chaudhary SK, et al. Profile of the 2016 dengue outbreak in Nepal. BMC Res Notes. 2018;11(1):423–423. 29970132 10.1186/s13104-018-3514-3 PMC6029055

[44] Chang C-J, Chen CS, Tien C-J, et al. Epidemiological, clinical and climatic characteristics of dengue fever in Kaohsiung City, Taiwan with implication for prevention and control. PLoS One. 2018;13(1):e0190637. 29293624 10.1371/journal.pone.0190637 PMC5749826

[45] Ooi EE, Goh KT, Chee Wang DN. Effect of increasing age on the trend of dengue and dengue hemorrhagic fever in Singapore. Int J Infect Dis. 2003;7(3):231–232. 14563228 10.1016/s1201-9712(03)90057-9

[46] Byrne AB, Gutierrez GF, Bruno A, et al. Age-associated differences in clinical manifestations and laboratory parameters during a dengue virus type 4 outbreak in Argentina. J Med Virol. 2018;90(2):197–203. 28941278 10.1002/jmv.24952

[47] Wangdi K, Clements ACA, Du T, et al. Spatial and temporal patterns of dengue infections in Timor-Leste, 2005-2013. Parasit Vectors. 2018;11(1):9. 29301546 10.1186/s13071-017-2588-4 PMC5755460

[48] Ehelepola ND, Ariyaratne K, Buddhadasa WM, et al. A study of the correlation between dengue and weather in Kandy City, Sri Lanka (2003 -2012) and lessons learned. Infect Dis Poverty. 2015;4:42. 26403283 10.1186/s40249-015-0075-8 PMC4581090

[49] Debnath F, Ponnaiah M, Acharya P. Dengue fever in a municipality of West Bengal, India, 2015: An outbreak investigation. Indian J Public Health. 2017;61(4):239–242. 29219127 10.4103/ijph.IJPH_309_16

[50] Sharmin S, Glass K, Viennet E, et al. Geostatistical mapping of the seasonal spread of under-reported dengue cases in Bangladesh. PLoS Negl Trop Dis. 2018;12(11):e0006947. 30439942 10.1371/journal.pntd.0006947 PMC6264868

[51] Wilder-Smith A, Gubler DJ. Geographic expansion of dengue: the impact of international travel. Med Clin North Am. 2008;92(6):1377–1390., x. 19061757 10.1016/j.mcna.2008.07.002

[52] Bostan N, Javed S, Nabgha EA, et al. Dengue fever virus in Pakistan: effects of seasonal pattern and temperature change on distribution of vector and virus. Rev Med Virol. 2017;27(1):e1899. 10.1002/rmv.1899 27597296

[53] Gomes AF, Nobre AA, Cruz OG. Temporal analysis of the relationship between dengue and meteorological variables in the city of Rio de Janeiro, Brazil, 2001-2009. Cad Saude Publica. 2012;28(11):2189–2197. 23147960 10.1590/s0102-311x2012001100018

[54] Reinhold JM, Lazzari CR, Lahondère C. Effects of the environmental temperature on Aedes aegypti and Aedes albopictus mosquitoes: A Review. Insects. 2018;9(4):158. 30404142 10.3390/insects9040158 PMC6316560

[55] Xiao FZ, Zhang Y, Deng YQ, et al. The effect of temperature on the extrinsic incubation period and infection rate of dengue virus serotype 2 infection in Aedes albopictus. Arch Virol. 2014;159(11):3053–3057. 24990415 10.1007/s00705-014-2051-1

[56] Teurlai M, Menkes CE, Cavarero V, et al. Socio-economic and climate factors associated with dengue fever spatial heterogeneity: a worked example in New Caledonia. PLoS Negl Trop Dis. 2015;9(12):e0004211. 26624008 10.1371/journal.pntd.0004211 PMC4666598

[57] Acharya BK, Cao C, Lakes T, et al. Modeling the spatially varying risk factors of dengue fever in Jhapa district, Nepal, using the semi-parametric geographically weighted regression model. Int J Biometeorol. 2018;62(11):1973–1986. 30182200 10.1007/s00484-018-1601-8

[58] Troyo A, Fuller DO, Calderón-Arguedas O, et al. Urban structure and dengue fever in Puntarenas, Costa Rica. Singap J Trop Geogr. 2009;30(2):265–282. 20161131 10.1111/j.1467-9493.2009.00367.x PMC2743112

[59] Araujo RV, Albertini MR, Costa-da-Silva AL, et al. Sao Paulo urban heat islands have a higher incidence of dengue than other urban areas. Braz J Infect Dis. 2015;19(2):146–155. 25523076 10.1016/j.bjid.2014.10.004 PMC9425226

